# Improvement of Transparencies and Mechanical Properties of Poly(cyclohexylene dimethylene cyclohexanedicarboxylate) Parts Using a Compounding Nucleating Agent to Control Crystallization

**DOI:** 10.3390/ma12040563

**Published:** 2019-02-14

**Authors:** Bei Su, Ying-Guo Zhou

**Affiliations:** School of Materials Science and Engineering, Jiangsu University of Science and Technology, Zhenjiang 212003, Jiangsu, China; subei_2005@126.com

**Keywords:** poly(cyclohexylene dimethylene cyclohexanedicarboxylate) (PCCE), nucleating agent, transparency, mechanical performance, crystallization kinetics, crystallization morphology

## Abstract

Poly(cyclohexylene dimethylene cyclohexanedicarboxylate) (PCCE) is a kind of copolyester polymer with excellent toughness and outstanding flexibility. However, the opacity caused by crystallization limits the widespread application of PCCE in products that have transparency requirements. The effects of 1,3:2,4-Di-p-methylbenzylidene sorbitol (MDBS) on the crystallization behavior, transparency, and mechanical properties of a PCCE melt were investigated via differential scanning calorimetry (DSC), spectrophotometry, and tensile testing. The results suggest that the transparency and mechanical properties of PCCE drastically improve and that its crystallization behaviors are obviously influenced by the addition of MDBS. PCCE with 0.6 wt% MDBS was then selected as a representative sample, and its thermal behavior and crystal morphology were further investigated by DSC, hot-staged polarizing microscopy (HSPLM), and scanning electron microscopy (SEM). The quantitative results suggest that, compared to neat PCCE resin, PCCE/MDBS has a lower isothermal and nonisothermal crystallization activation energy, which indicates a rapid crystallization process. The results also show that, compared to the pure PCCE melt, the PCCE/MDBS melt experiences a greater increase in the number of crystals and a greater decrease in the crystal size during cooling. The acceleration of the crystallization process and reduction in crystal size can be both attributed to the nucleation effect of the MDBS. In conclusion, because the addition of the nucleating agent improves the transparency and tensile properties of PCCE by adjusting and controlling its thermal and crystallization behaviors, the proposed technique of using a compounding nucleating agent to control crystallization is therefore suitable for PCCE.

## 1. Introduction

Transparency is one of the most important focuses in polymer engineering, because it is a requirement for many applications, such as packaging, electronic instruments, and optical apparatuses. It is known that the transparencies of amorphous polymers are generally better than those of crystalline materials. Therefore, crystallization is often considered an important factor affecting the transparencies of polymers. Semicrystalline polymers can be either transparent or opaque, and, aside from the material composition, this behavior is often dependent on the detailed crystallization process. It is well known that nucleating agents (NAs) can increase the speed of crystallization and diminish the crystal sizes in semicrystalline polymers. Hence, NAs are frequently used in real polymer processing because adjusting the crystal size and controlling the crystallinity [1,2,3,4,5,6,7,8] have been conventional methods to modify the mechanical properties of semicrystalline polymers. Many NAs including organic and inorganic micro- or nanoparticle/fibers, such as organoclay [9], montmorillonite [10,11], nano-SiO_2_/silica [12,13], carbon nanotubes [14,15], talc [16,17], and various types of fibers [18,19,20], have been reported to improve the crystallization behaviors of many semicrystalline polymers, such as isotactic polypropylene (iPP), polyoxymethylene (POM), polyamide (PA), and polyethylene 2,6-naphthalate (PEN); these improvements in crystallization behaviors have led to substantial improvements in the mechanical properties of the matrices. However, commonly used NAs have difficulty improving the transparency of the matrix because of their relatively weak nucleation effect and inappropriate crystallization behavior. Sorbitol products, such as dibenzylidene sorbitol (DBS) and methylbenzylidene sorbitol (MDBS), have a special chemical structure of butterfly-shaped groups that can self-organize into a nanofibrillar network [21]. The formed nanofibrils have high surface areas that can promote heterogeneous nucleation by inducing crystallizable macromolecular chains into numerous small spherulites [22], which were found to facilitate the transparency of isotactic polypropylene (iPP) [23,24].

Poly(cyclohexylene dimethylene cyclohexanedicarboxylate) (PCCE) is a kind of copolyester polymer [25] that can be used as a high-performance thermoplastic material in extruded and injection-molded components because of its excellent toughness, chemical resistance, and flexibility. For example, medical packaging is one of its representative applications. However, PCCE, which is a typical semicrystalline polymer, has a high crystallization ability; it can be crystallized under a cooling rate as high as 100 °C/min. The rapid crystallization and large crystal size result in PCCE products being either semitransparent or opaque once the thickness of the manufactured components exceeds a certain limit. In addition, the PCCE also exhibits a low mechanical strength and modulus. Both the transparency and mechanical properties limit the applications of PCCE products in a broader range of polymer engineering. Therefore, it is necessary and feasible to improve the transparency and mechanical properties of PCCE components by adjusting the crystallization behaviors of PCCE. However, to the best of our knowledge, there has been little research related to the macroscale mechanical, optical performances or microscale crystallization structures of micro- or nanoparticle/fiber-filled PCCE composites. This limitation in the literature has aroused our interest to explore the topic. In this study, an appropriate NA was selected and added to the PCCE melt, and the transparencies, mechanical properties, thermal behaviors, and crystallization behaviors of the filled PCCE composites were investigated. To avoid the possible influence of fabrication methods, extruded and injection-molded samples were both prepared and characterized. 

## 2. Experiment

### 2.1. Materials

The PCCE (Ecdel 9967, MFI: 4.0 g/10 min (230 °C, 21.6 N)) used in this study was a commercial product from Eastman Chemical Products Inc., Kingsburg, TN, USA. Before use, the PCCE was dried in an oven at 130 °C for 6 h to remove any excess moisture. The nucleating agent (NA) used in this study was 1,3:2,4-Di-p-methylbenzylidene sorbitol (MDBS), which have particle sizes in the range of 2~2.5 μm.

### 2.2. Sample Preparations

PCCE/MDBS composites with MDBS contents of 0.2 wt%, 0.4 wt%, 0.6 wt%, and 0.8 wt%, which were denoted PCCE/2MDBS, PCCE/4MDBS, PCCE/6MDBS, and PCCE/8MDBS, respectively, were prepared by two-step compounding. The composites were first mixed in a high-speed mixer (Hengfei, Suzhou, China) and then compounded using a twin-screw extruder (SHJ30, Giant, Nanjing, China) with a single-orifice die (Φ3 mm). The extrusion processing parameters was as follows: the temperature from the hopper to the die of the extruder were 165, 180, 195, and 185 °C, respectively, the speed of the screw rotation was 30 rpm, the speed of the take-up rolls was 0.5 m/s, and the temperature of take-up rolls was 50 °C. The extruded material was cooled naturally at the room conditions (25 °C) and then pelletized. For comparison, a neat PCCE sample was also processed via the same procedure. 

Two types of PCCE/MDBS samples were then fabricated by using a conventional injection molding machine (ZX-80T, ZhenDe Company, Guangzhou, China) and a laboratory-level single-screw extruder (Haake Rheomex 252p, Thermo Fisher Scientific, Karlsruhe, Germany) with a slit die (0.5 mm by 50 mm). Each injection-molded dog-bone sample has a volume of approximately 9.57 × 10^3^ mm^3^ and a thickness of 4 mm in accordance with the standard of ASTM-D638. The injection molding processing parameters were as follows: the injection machine melt temperature was 205 °C, the mold temperature was 65 °C, the injection pressure was 65 MPa, the injection time was 1.5 s, the packing pressure was 60 MPa, the packing time was 3 s, and the cooling time was 20 s. To obtain a moderate thickness of sheet during extrusion, the optimized extrusion parameters were determined after a series of screening procedures. The temperatures, from the hopper to the die of the extruder, were 170, 185, 200, and 190 °C, and the draw ratio (DR, which is the ratio of the rolling rate and the extrusion rate) was approximately 8.3, which produced an extruded sheet with a thickness of approximately 0.1 mm. 

### 2.3. Optical Performance Tests

The optical properties of each PCCE/MDBS injection-molded and extruded sample were analyzed at 25 °C using a spectrophotometer (UV-3600 with an MPC-3100 multipurpose sample compartment, Shimadzu, Tokyo, Japan) operated in the wavelength range from 400 to 700 nm. At least five scans were collected from each group of samples to avoid possible experimental deviations and to guarantee data reproducibility.

### 2.4. Tensile Tests

The tensile tests were performed using a multiuse mechanical testing machine (MTS, Eden Prairie, MN, USA, Sintech 10/GL) at 25 °C with a displacement rate of 10 mm/min. The tensile test bars were obtained by either punching the bars from the middle of the extruded sheets or by directly using the injection-molded components. At least seven tensile bars were tested for each formulation, and the mean value and range of the modulus of elasticity (Young’s modulus), ultimate tensile strength, and strain-at-break for each sample were calculated. In this study, the tensile strengths were nominal, and they were determined by dividing the loads by the original cross-sectional area. 

### 2.5. Thermal Analyses

Thermal analyses were performed with a differential scanning calorimetry (DSC, DSC-8000, Perkin-Elmer, Shanghai, China) apparatus. All DSC samples were cut from the middle of the extruded sheets, and their weights were strictly limited in the range of 4.9~5.1 mg to avoid any possible influence of the sample weight. The temperature of the DSC apparatus was also carefully calibrated with indium prior to testing. Samples were loaded in the DSC at 25 °C, and liquid nitrogen was used as the cooling fluid. The DSC tests can be classified into three groups. 

In the first testing group, the five PCCE/MDBS specimens (PCCE, PCCE/2MDBS, PCCE/4MDBS, PCCE/6MDBS, and PCCE/8MDBS) were heated to 240 °C and held isothermally for 5 min to eliminate any prior thermomechanical history. Then, the samples were cooled to 25 °C at 10 °C/min. 

The second testing group was used to analyze the isothermal crystallization process of PCCE/6MDBS. The samples were rapidly heated to 240 °C, held isothermally for 5 min, and then cooled to the designated crystallization temperatures (*T*_c_) at 150 °C/min for isothermal crystallization; the nine different *T*_c_ values used in this study were 164, 166, 168, 170, 172, 174, 176, 178, and 180 °C. Finally, the samples were cooled to 25 °C at 10 °C/min. 

The third testing group was used to analyze the nonisothermal crystallization process of PCCE/6MDBS. The samples were heated from 25 °C to 240 °C rapidly, held isothermally for 5 min, and then cooled to 25 °C with the following cooling rates: 2, 5, 10, 20, 30, 50, and 100 °C/min. The selected cooling rates were varied as much as possible to obtain a greater amount of information regarding the practical processing of the samples. 

### 2.6. Morphological Observations

The selected PCCE/6MDBS and neat PCCE samples were observed by hot-staged polarizing light microscopy (HSPLM, BX61, OLYMPUS, Beijing, China) with the following heating/cooling procedure: the samples were heated from 25 °C to 240 °C at 10 °C/min, held isothermally for 5 min, and then cooled to 25 °C at 10 °C/min. Each specimen was cut from the cross-section at the middle of the molded tensile bar via an ultrathin sectioning technique.

The morphologies of the PCCE/6MDBS and neat PCCE samples were examined via a scanning electron microscopy (SEM JSM-6480, JEOL, Tokyo, Japan) device with an accelerating voltage of 20 kV. Each SEM specimen was taken from the cross-section at the middle of the injection-molded tensile bar, which was frozen, and then the specimen was sputtered with a thin layer of gold. To better characterize the morphological differences between the molded PCCE and PCCE/6MDBS samples, the fractured surfaces were etched using a solution of dichloromethane for approximately 1 min at 25 °C and then flushed with high-pressure water. The etched samples were also observed via SEM after they were dried and gold-sputtered.

## 3. Results and Discussion

### 3.1. Comparison among PCCE/MDBSs

#### 3.1.1. Transparencies

**Figure 1:**
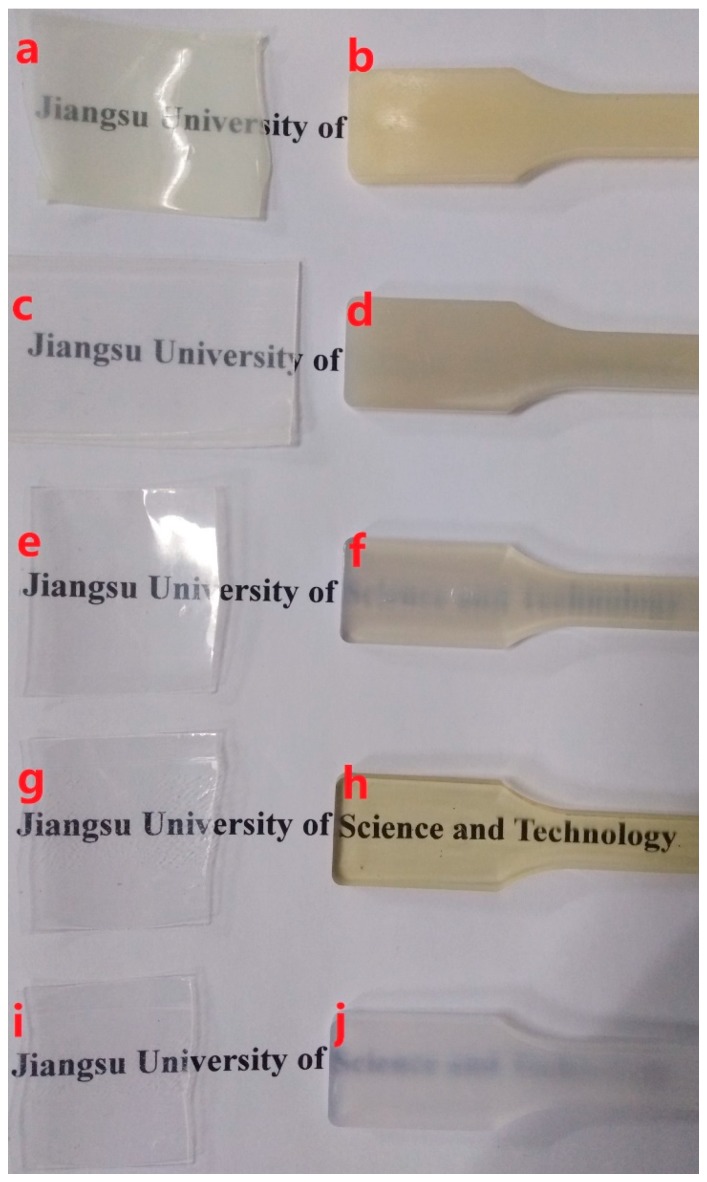
Visual comparison of the (a, c, e, g, and i) extruded and (b, d, f, h, and j) injection-molded PCCE/MDBS samples with different amounts of MDBS (a and b show the pure PCCE sample; c and d show the PCCE/2MDBS sample; e and f show the PCCE/4MDBS sample; g and h show the PCCE/6MDBS sample; and i and j show the PCCE/8MDBS sample).

Figure 1 shows an illustrative comparison of the transparencies of the extruded and injection-molded samples of the PCCE/MDBS composites. Figure 1 shows that the extruded samples (a, c, e, g, and i) with thicknesses of 100 µm are all transparent, although a slight difference in transparency can be found among the samples. However, the transparencies of the injection-molded PCCE/MDBS samples with thicknesses of 4 mm (b, d, f, h, and j) obviously vary, and the text below the sample becomes clearer and then hazier with respect to increases in the MDBS content. This phenomenon indicates that the transparencies of PCCE/MDBS composites depend not only on their thicknesses, but also on their compositions. Taken together, the PCCE/6MDBS samples, both extruded and injection-molded, are the most transparent samples of the five PCCE/MDBS composites.

**Figure 2:**
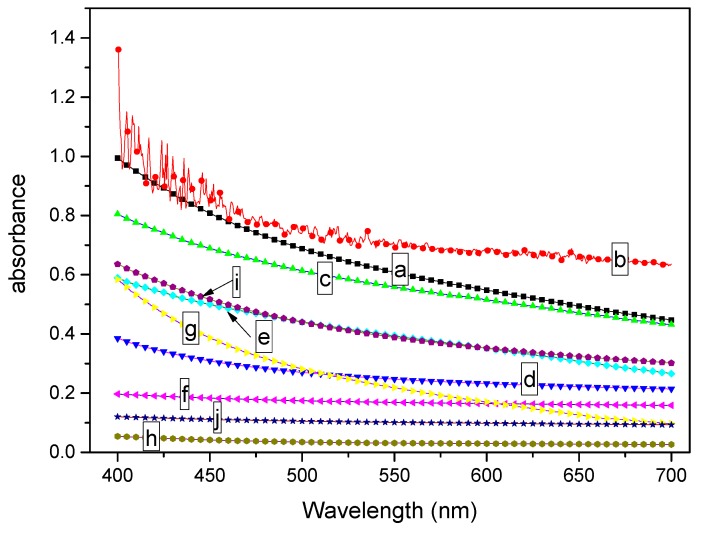
Light absorbance comparisons of the (a, c, e, g, and i) extruded and (b, d, f, h, and j) injection-molded PCCE/MDBS samples with different amounts of MDBS (a and b show the pure PCCE sample; c and d show the PCCE/2MDBS sample; e and f show the PCCE/4MDBS sample; g and h show the PCCE/6MDBS sample; and i and j show the PCCE/8MDBS sample) in 1mm thickness.

To quantitatively describe the effect of MDBS on the transparency of PCCE, the samples shown in Figure 1 were analyzed using a spectrophotometer, and the light absorbance results are shown in Figure 2. It must be noted that the reported absorbance in Figure 2 has a unit thickness (1 mm) result. It can be seen from Figure 2 that the absorbance value is different between the extruded and the injection-molded samples for each formulation of the PCCE/MDBS composites, which indicated effect of the fabrication of the sample on the optical performance. However, compared to pure PCCE samples, regardless of whether they were extruded or injection-molded, the absorbance values of the PCCE/MDBS samples were all higher, and their absorbance values exhibited a decreasing and then increasing trend with increasing MDBS content. These results are consistent with the visual comparison results shown in Figure 1, which indicates that the existence of MDBS improves the transparencies of PCCE components. In addition, the transparency of PCCE/MDBS decreases from its optimum value when the content of MDBS exceeds 0.6 wt%. 

#### 3.1.2. Mechanical Properties

**Figure 3:**
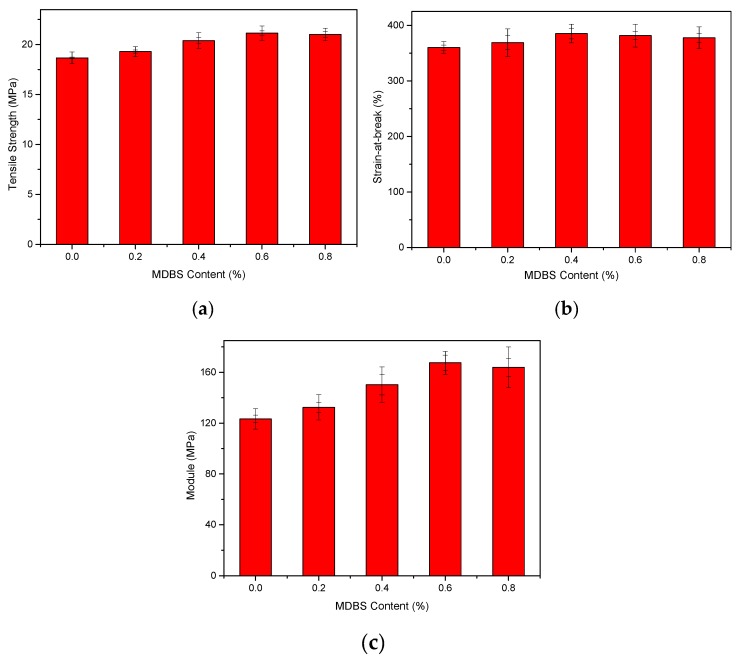
Experimental results of the (**a**) tensile strengths, (**b**) strain-at-break values, and (**c**) moduli of the PCCE/MDBS injection-molded samples (the two error bars express the biggest and the smallest error, respectively).

**Figure 4:**
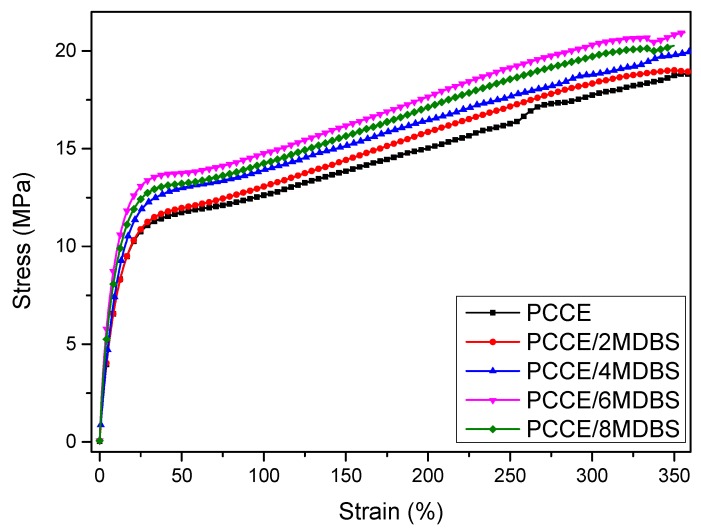
The tensile stress-strain curves of the PCCE/MDBS extruded samples.

Figure 3 shows the tensile strengths, strain-at-break values, and moduli of the injection-molded samples at a displacement rate of 10 mm/min with different MDBS contents. Figure 3 shows that the mechanical properties of PCCE are lower than those of the PCCE/MDBS composites. Furthermore, the tensile properties of the PCCE/MDBS composite are also very sensitive to the MDBS content. As the MDBS content increases, the tensile strengths and Young’s moduli of the samples displayed a trend of first increasing and then decreasing. It is worth noting that, regardless of the content of MDBS, the strain-at-break values of the PCCE/MDBS composites were also higher than that of pure PCCE resin, which indicates that the toughness does not worsen with the existence of the MDBS. It can be concluded that the MDBS addition improves the mechanical properties of the PCCE components, and PCCE/6MDBS has the best mechanical properties among all five PCCE/MDBS composites. It can also be seen from Figure 3 that, compared to the pure PCCE resin, the yield strength, tensile strength, strain-at-break, and tensile modulus of the PCCE/6MDBS sample increased by approximately 20%, 9%, 6%, and 30%, respectively. For clarification, as shown in Figure 4, four representative stress-strain plots are selected for comparison among the different PCCE/MDBS extruded samples. It can be observed from Figure 3 and Figure 4 that the fabrication methods used to produce the tensile testing samples, either extrusion or injection-molding, have no substantial impact on the stress–strain behaviors of the samples. For the PCCE/MDBS composite parts, no matter what the MDBS content is, yielding can be observed at approximately 20% strain. However, the yield strengths obviously increase with increasing MDBS content when the MDBS content does not exceed 0.6 wt%. Furthermore, after yielding, all five PCCE/MDBS samples exhibit strain-hardening, and the nominal stress continues to increase with increasing strain until fracturing. The effect of the MDBS content on the nominal stress can also be observed. At the same strain, the higher the content of MDBS in the PCCE/MDBS sample is within the range of 0~0.6 wt%, the higher the nominal stress. Therefore, from Figure 3 and Figure 4, a conclusion can be drawn that the mechanical properties of PCCE elastomers can be improved by the addition of MDBS and that the best mechanical properties can be obtained at an MDBS content of 0.6 wt%. In general, regardless of the case, the material properties are often one of the most important considerations for potential applications. Hence, the incorporation of MDBS into PCCE is believed to have remarkable importance, and PCCE/6MDBS is possibly the best choice when both transparency and mechanical properties are required.

#### 3.1.3. Crystallization Behaviors

**Figure 5:**
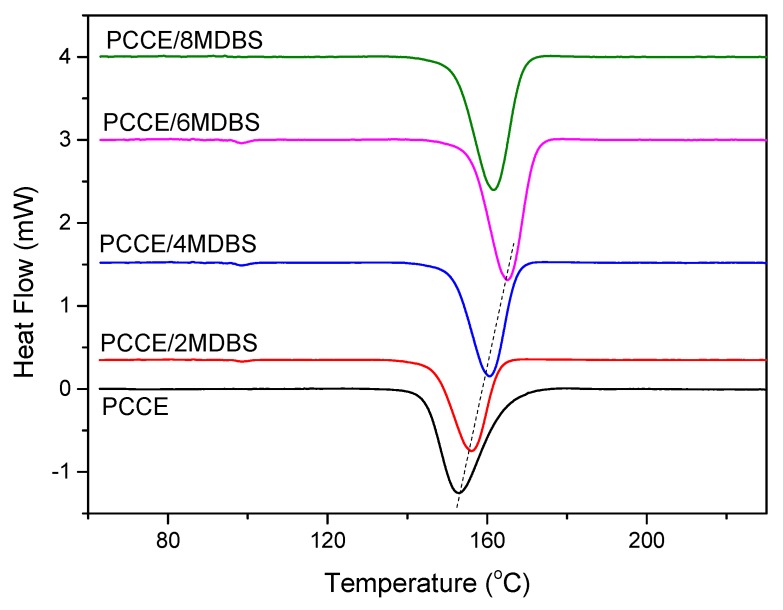
DSC thermograms of the PCCE/MDBS samples at a cooling rate of 10 °C/min.

It is well known that the different properties of polymer parts often depend on their microstructures. As discussed before, PCCE is a typical semicrystalline material with high crystallization ability. The crystallization behavior of PCCE, which may produce different properties, is always the key to understanding its microstructure. In general, crystallization behavior mainly include crystallization kinetics and crystallization morphologies. Thermal analyses are carried out to understand the different crystallization kinetics of PCCE/MDBS composites. The DSC thermograms of the PCCE samples with different amounts of MDBS at a cooling rate of 10 °C/min are shown in Figure 5. A crystallization peak is observed for all five tested PCCE/MDBS samples; however, these peaks appear at different times and have different forms when the samples have different amounts of MDBS. Similar to the effects of the MDBS content on transparency and mechanical properties, in the range of 0~0.6 wt%, the higher the content of MDBS is, the quicker the peak appears and the narrower its shape. This result indicates an obvious acceleration effect of the crystallization process of MDBS in the PCCE melts. However, the crystallization rate decreases when the content of MDBS exceeds 0.6 wt%. These results also correspond to the peak temperatures shown in Figure 5. Therefore, 0.6 wt% is the optimal MDBS content for improving the transparency and mechanical properties of PCCE by adjusting the crystallization behavior into the most suitable state.

**Figure 6:**
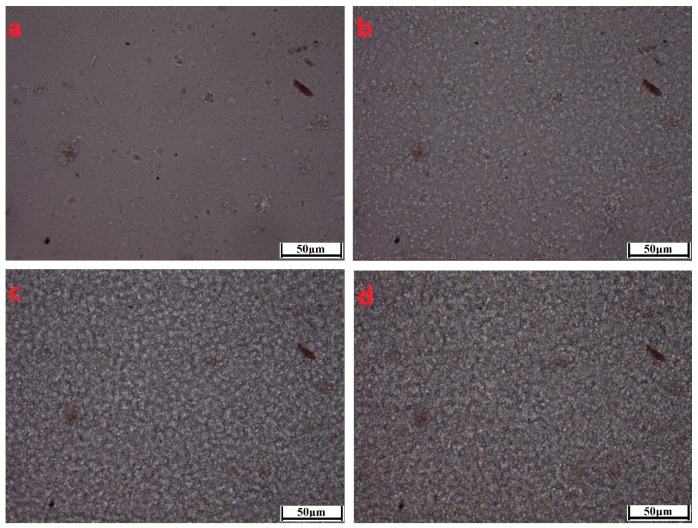
HSPLM images of the neat PCCE crystallization at a cooling rate of 10 °C/min. The recorded temperatures in subgraphs (**a**–**d**) correspond to 176 °C, 174 °C, 172 °C, and 170 °C, respectively.

**Figure 7:**
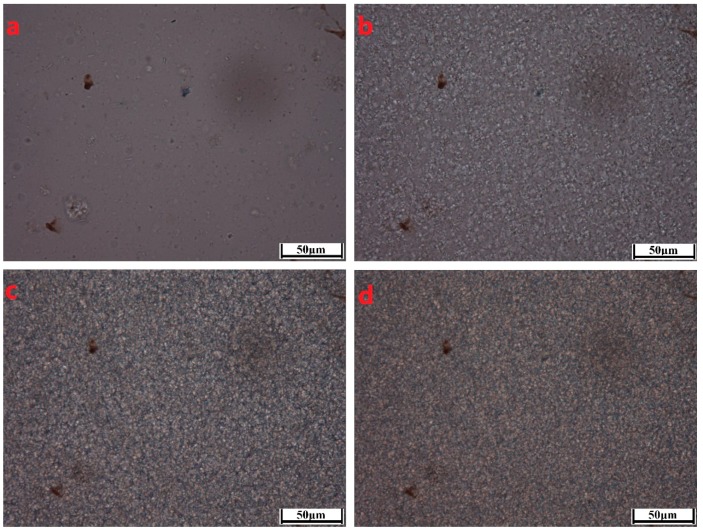
HSPLM images of the PCCE/6MDBS crystallization at a cooling rate of 10 °C/min. The recorded temperatures in subgraphs (**a**–**d**) correspond to 178 °C, 176 °C, 174 °C, and 172 °C, respectively.

To better characterize the crystallization morphological evolutions of the PCCE/MDBS composites, two representative samples, neat PCCE and PCCE/6MDBS samples, were observed by HSPLM, and the results are shown in Figure 6 and Figure 7, respectively. The recorded temperatures in subgraphs (a), (b), (c), and (d) of Figure 6 and Figure 7 correspond to 176 °C, 174 °C, 172 °C, and 170 °C, and 178 °C, 176 °C, 174 °C, and 172 °C, respectively. The nucleations and crystal growths can be found for both PCCE and PCCE/6MBS samples from the two figures. However, compared to the pure PCCE crystallization process shown in Figure 6, the PCCE/6MDBS crystallization process shown in Figure 7 had earlier crystallization appearance, more nuclei, and smaller crystals. This phenomenon can be attributed to the nucleation effects of MDBS on the PCCE composites.

**Figure 8:**
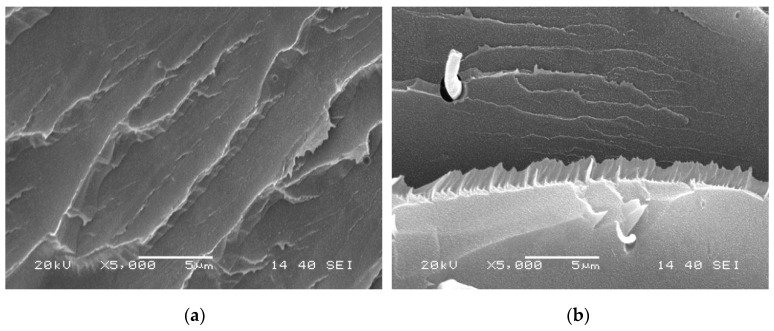
SEM images of the fracture surfaces of the (**a**) pure PCCE and (**b**) PCCE/6MDBS samples.

**Figure 9:**
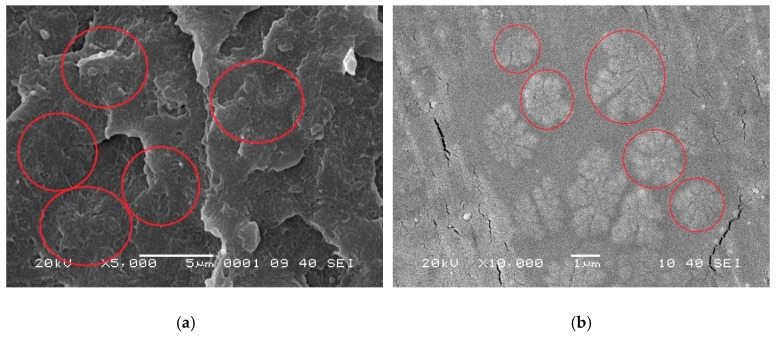
SEM images of the etched fracture surfaces of the (**a**) pure PCCE and (**b**) PCCE/6MDBS samples.

The boundaries of crystals shown in Figure 6 and Figure 7 are not possibly distinct enough to quantitatively compare the process of crystal growth owing to the small size and large number of crystals that appear almost simultaneously during the HSPLM observation. In particular, the existence of MDBS obviously accelerates the originally rapid crystallization process of PCCE. Hence, a qualitative conclusion of the nucleation effect of MDBS is drawn from the direct observation of the crystals via HSPLM. It is necessary to observe the morphology under a microscope with a higher magnification; SEM is thus the ideal method to further investigate the resulting morphological differences. A comparison of the fracture surfaces of pure PCCE and PCCE/6MDBS using SEM is shown in Figure 8. The fracture of PCCE/6MDBS shown in Figure 8b is more refined than that of PCCE shown in Figure 8a. The details of the crystals can also be further explored by SEM observations of the etched PCCE and PCCE/6MDBS samples shown in Figure 9. It can be observed from Figure 9 that the crystals in the PCCE/6MDBS sample are obviously smaller and more uniform than those in the pure PCCE sample. Compared to the crystal size of 5 µm of the pure PCCE shown in Figure 9a, the crystals of PCCE/6MDBS are approximately 1–2 µm, as indicated in Figure 9b. These quantitative results indicate that the addition of MDBS particles can produce fine crystals and promote nucleation in the PCCE melt.

Thus far, it has already been indicated that the crystallization morphology of the PCCE/6MDBS is different from that of the pure PCCE resin. However, to quantify the crystallization process parameters, it is still necessary to further investigate the crystallization kinetics of the PCCE/MDBS samples to understand the mechanism responsible for improving their transparency and mechanical properties, which may result from the effect of MDBS on the crystallization behaviors, as previously discussed. For the sake of brevity, PCCE/6MDBS was still selected as a representative sample, and a careful comparison of the crystallization kinetics parameters between PCCE/6MDBS and pure PCCE was made. In our previous work [25], a series of parameters of pure PCCE crystallization, such as the crystallization active energy and crystallization rate parameter, were obtained after careful investigation of the isothermal and nonisothermal crystallization kinetics of a pure PCCE melt. Therefore, it is feasible to compare these parameters with the reported results. The reference dates of neat PCCE in the following sections are no longer marked. 

### 3.2. Crystallization Kinetics of PCCE/6MDBS

#### 3.2.1. Isothermal Crystallization Kinetics Analyses

**Figure 10:**
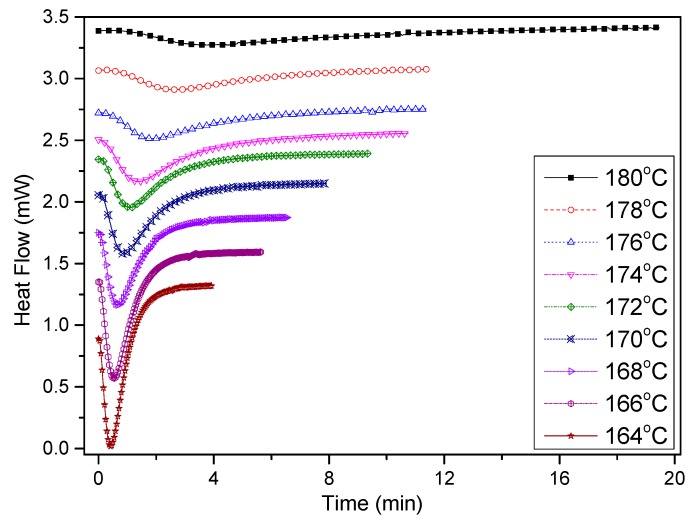
Heat flow versus time of PCCE/6MDBS at specified crystallization temperatures obtained via DSC.

Figure 10 shows the heat flow curves of the PCCE/6MDBS samples in the isothermal crystallization process, which have similar shapes to those of pure PCCE. As shown in Figure 10, a crystallization peak can be observed for all tested samples. However, the appearance times of the peaks are different with respect to different crystallization temperatures *T*_c_. The lower the value of *T*_c_ is, the quicker the crystallization peak appears and the narrower its shape. This phenomenon indicates that the crystallization rate is relatively faster at low values of *T*_c_. Therefore, the isothermal crystallization process of PCCE/6MDBS is temperature dependent.

**Figure 11:**
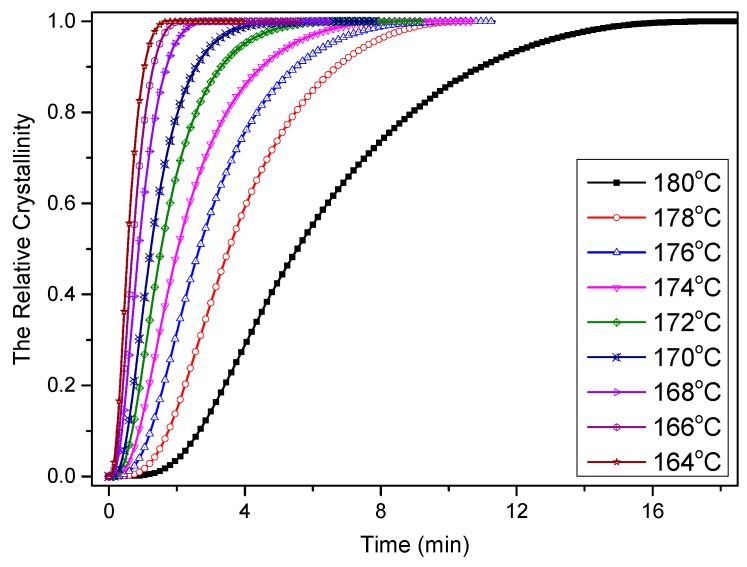
Relative crystallinity versus time of PCCE/6MDBS at specified crystallization temperatures obtained via DSC.

From the heat flow curves shown in Figure 10, the relative crystallinity values of the samples can be calculated by the following expression [26]:

(1) $$\alpha (t)=\int_{t_{0}}^{t}\frac{dH}{dt}dt/\int_{t_{0}}^{\infty }\frac{dH}{dt}dt$$

where *α*(*t*) is the evolution in the relative crystallinity value with respect to time *t*, *t*_0_ and *t* are the time at which the crystallization begins and the measurement is taken, respectively, and d*H*/d*t* is the heat flow rate in the crystallization stage. The evolution in the relative crystallinity with respect to time is presented in Figure 11, and it was found that all curves exhibited similar sigmoidal shapes. 

The relative crystallinity is often analyzed using the following Avrami expression [27]:

(2) $$\alpha (t)=1-\exp(-K(T)t^{n})$$

where *n* is the Avrami exponent and *K* is the crystallization rate parameter at the isothermal crystallization temperature *T*. The Avrami model (Equation (2)) can be easily rewritten as the following logarithmic form after a mathematical transformation:

(3) log(−ln(1−α(t)))=log(K(T))+nlog(t)

**Figure 12:**
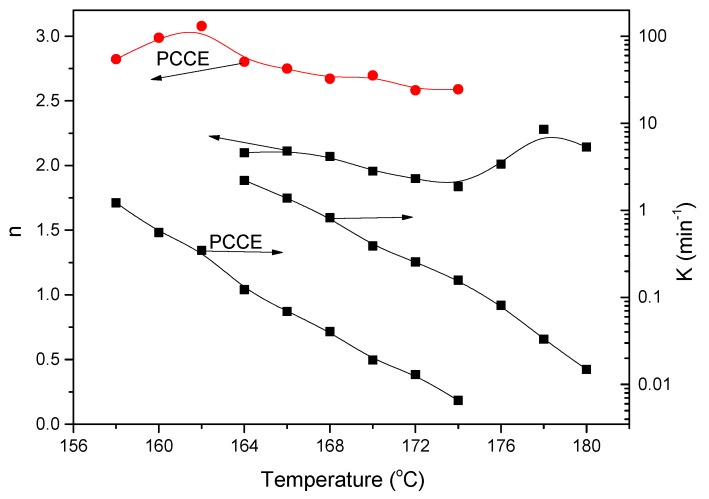
Comparison of the Avrami exponent *n* and crystallization rate parameter *K* between the pure PCCE and PCCE/6MDBS samples.

Applying Equation (3), the values of *n* and *K* can be linearly fitted, and the obtained results are shown in Figure 12. The values of *n* for PCCE/6MDBS range from 1.84 to 2.28, which depends on the *T*_c_; this indicates that the spherulite growth mode of PCCE/6MDBS is mainly in a two-dimensional orientation with heterogeneous nucleation. The values of *K* increase as *T*_c_ decreases, which suggests a rapid crystallization process. Compared to the results of neat PCCE, the values of *n* for PCCE/6MDBS are relatively small at the same *T*_c_, which indicates that the MDBS particles act as heterogeneous nuclei in the primary crystallization stage. Moreover, compared to the neat PCCE, the *K* values of PCCE/6MDBS are larger, which suggests that the incorporation of the MDBS particles accelerates the crystallization process of the PCCE matrix.

#### 3.2.2. Isothermal Crystallization Activation Energy of PCCE/6MDBS 

**Figure 13:**
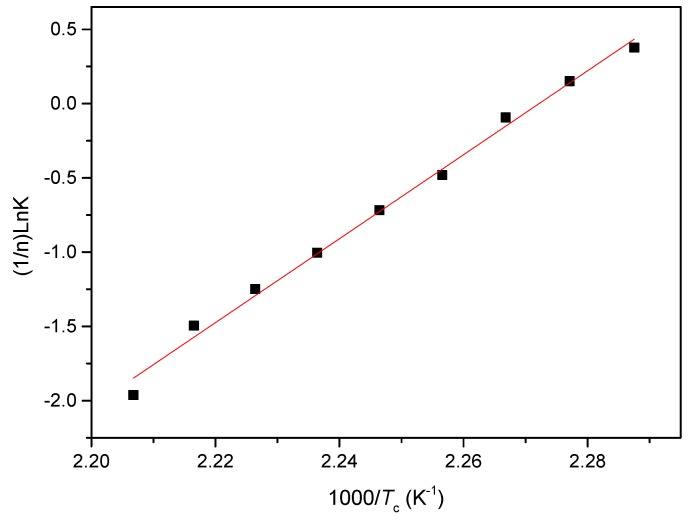
Plot of (1/n)lnK versus 1/*T*_c_ for the acquisition of the isothermal crystallization activation energy of PCCE/6MDBS.

Based on the assumption that the isothermal crystallization process of PCCE/6MDBS is thermally activated, the relationship between the crystallization rate parameter *K* and the crystallization activate energy *ΔE* can be expressed as the following logarithm form [28,29,30]:

(4) $$\frac{1}{n}\cdot \ln K=\ln K_{0}-\frac{\Delta E}{R\cdot T_{c}}$$

where *K*_0_ is the temperature-independent pre-exponential parameter and *R* is the gas constant. The plot of (1/*n*)ln*K* versus 1/*T*_c_ is shown in Figure 13 and the slope coefficient of the least square fitted line can be used to calculate the *ΔE*. From Figure 13, the value of *ΔE* for the isothermal crystallization process of PCCE/6MDBS can be determined as −235.1kJ/mol. Compared to the neat PCCE of −203.1 kJ/mol, the crystallization activation energy of PCCE/6MDBS is lower. A conclusion can be drawn that the MDBS makes the molecular chains of PCCE easier to crystallize in the isothermal crystallization process. 

#### 3.2.3. Nonisothermal Crystallization Activation Energy of PCCE/6MDBS

**Figure 14:**
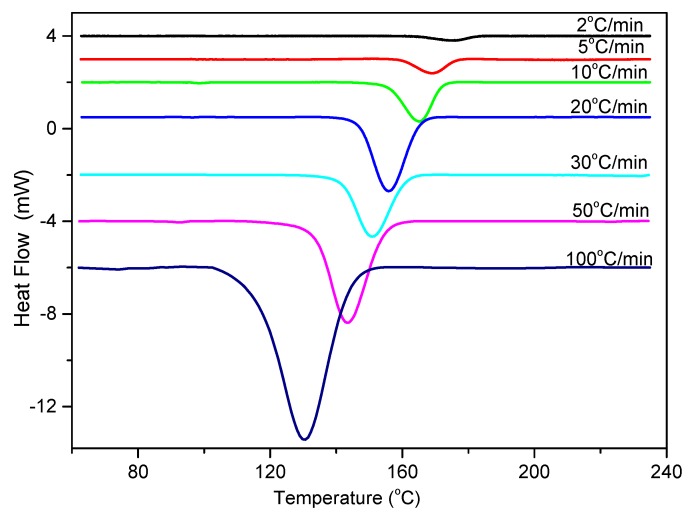
DSC thermograms of PCCE/6MDBS at the indicated cooling rates.

**Figure 15:**
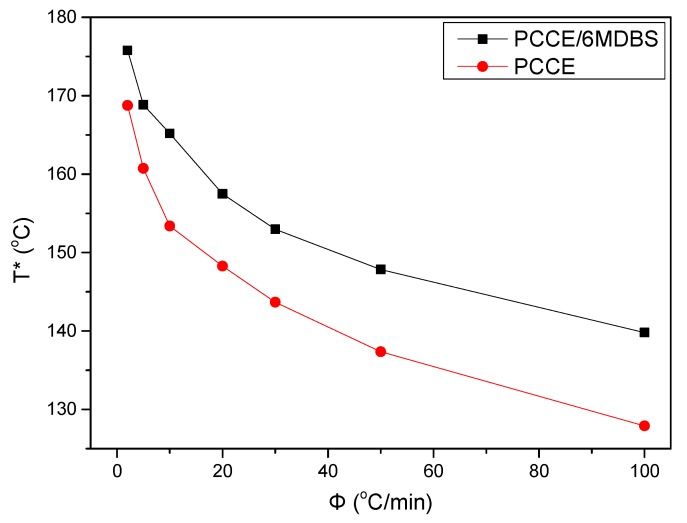
Comparison of the crystallization peak temperature between pure PCCE and PCCE/6MBS at the indicated cooling rates.

**Figure 16:**
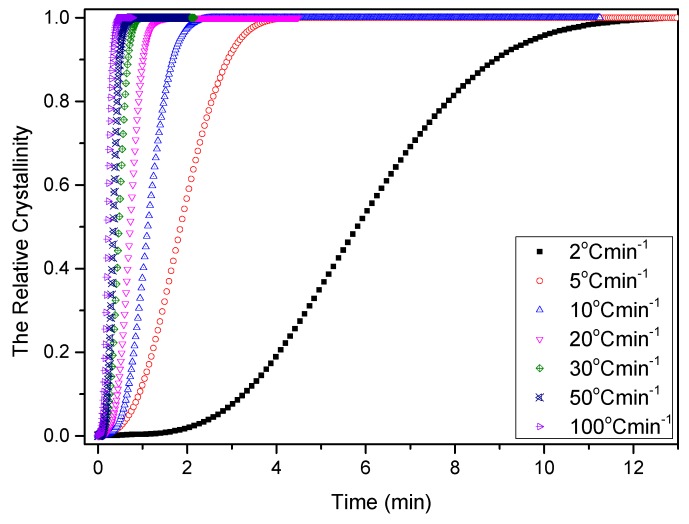
Relative crystallinity versus time of PCCE/6MDBS at the indicated cooling rates.

Temperature variation in real processing conditions is generally close to nonisothermal processes. Therefore, it is still necessary to investigate the nonisothermal crystallization process of PCCE/6MDBS. Figure 14 shows the heat flow curves of PCCE/6MDBS at various cooling rates, *Φ*. It can be seen from Figure 14 that the crystallization peak temperature (T*) is obviously different at different values of *Φ*. After a correction of the thermal lag [31,32,33,34], which is necessary because of the widely varied range of *Φ* used in this study, the results of T* at different values of *Φ* can be obtained, as shown in Figure 15. It can be found that T* increases as *Φ* decreases. Compared to the neat PCCE, the T* of PCCE/6MDBS is approximately 7~12 °C higher, which depends on the different *Φ* values; this result indicates that the maximum crystallization rate of the PCCE/6MDBS composite occurs earlier in the PCCE/6MDBS sample than in the neat PCCE sample. Furthermore, the temperature can be easily converted into of the nonisothermal crystallization processing time, and the evolution in the relative crystallinity versus time can be calculated based on Equation (1) and Figure 14, as depicted in Figure 16. All curves in Figure 16 have similar sigmoidal shapes. It can also be clearly determined from Figure 16 that the crystallization time lasts only less than 0.5 min at a cooling rate of 100 °C/min, whereas the crystallization time is as high as 12 min at a cooling rate of 2 °C/min. 

**Figure 17:**
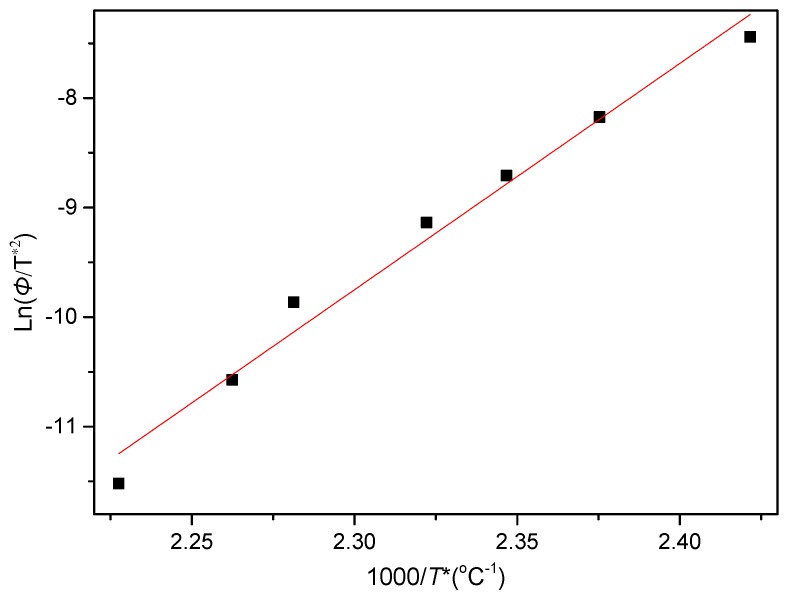
Plot of ln(*Φ*/T*^2^) versus 1/T* for the acquisition of the nonisothermal crystallization activation energy of PCCE/6MDBS.

It is well known that polymers with high crystallization rates generally possess low crystallization activation energies *ΔE*. Therefore, to quantitatively compare the differences between the PCCE and PCCE/6MDBS samples, the *ΔE* of PCCE/6MBS is calculated with a method that is similar to that used to obtain the result of PCCE, which was based on the generalized Kissinger method [35]. The *ΔE* values of the various *Φ* in the nonisothermal crystallization process of the PCCE/6MDBS composite can be calculated with the following expression.

(5) $$\frac{d(\ln(\varphi /T^{*2}))}{d(1/T^{*})}=-\frac{\Delta E}{R}$$

where T* is the peak temperature shown in Figure 15. The plot of ln(*Φ*/T*^2^) versus 1/T* is then presented in Figure 17. The *ΔE* of PCCE/6MDBS can be determined as −171.9 kJ/mol from the slope of the fitted line. Compared to the *ΔE* value of neat PCCE in the nonisothermal crystallization process, which is −146 kJ/mol, a decrease of 25.9 kJ/mol for the PCCE/6MDBS is obtained; this result indicates that the MDBS makes the molecular chains of PCCE easier to crystallize in the nonisothermal crystallization process.

According to the above discussion, the improvement in the optical and mechanical properties resulting from the incorporation of MDBS is attributed to the variation in the crystallization behaviors, and then the presented approach of compounding NA to control crystallization is concluded to be a suitable and efficient technique for PCCE modification. In addition, although the MDBS particle size is on a microscale, the nanofibers formed by the self-organization of MDBS work better as an NA [36,37]. Compared to generalized nanoparticles used in polymer engineering [11,38,39], from the view of industrial production, the microscale MDBS is more easily dispersed in the matrix; nanoparticles dispersed in polymer matrices are prone to aggregation, due to their small particle sizes, large specific surface areas, and high surface energies [40,41,42,43]. Therefore, microscale MDBS is more convenient for processing and production. It is also well known that the microstructure of a material determines its performance, which then decides its application. Therefore, for PCCE elastomers, if the transparency and/or tensile properties are of primary concern, the addition of MDBS in moderation can be considered a practical and efficient method. It is also worth investigating the applicability of this special kind of NA for other kinds of semicrystalline polymers and/or their blends to further understand the property improvements offered by controlling crystallization.

## 4. Conclusions

In this study, the effect of MDBS on the transparency, mechanical performance, and crystallization behavior of a PCCE melt were carefully investigated using spectrophotometry, tensile testing, DSC, HSPLM, and SEM. The results showed that the existence of MDBS can improve the transparency, tensile strength, toughness, and modulus of PCCE, which results from the accelerated crystallization process and the reduction in crystal size. The optimal MDBS content of 0.6 wt% was also determined by the comparison of the transparencies and tensile performances among the five PCCE/MDBS composites: pure PCCE, PCCE/0.2wt%MDBS, PCCE/0.4wt%MDBS, PCCE/0.6wt%MDBS, and PCCE/0.8wt%MDBS. Furthermore, compared to the crystallization morphology and kinetics of pure PCCE, the PCCE/0.6wt%MDBS was found to have smaller crystal sizes and more nucleation, which was deduced from the direct morphological observations and its lower crystallization activation energy, which provided a quantitative decrease of 32.0 kJ/mol in the isothermal crystallization activation energy, a decrease of 25.9 kJ/mol in the nonisothermal crystallization activation energy, and a smaller Avrami exponent, which indicates that the nucleation mode changed from homogeneous nucleation to heterogeneous nucleation. Accordingly, an effective way to improve the transparencies and mechanical behaviors of PCCE parts by using a nucleating agent to control crystallization was proposed and analyzed.
